# Social Organisation Predicts Lifespan in Mammals

**DOI:** 10.1002/ece3.73587

**Published:** 2026-04-28

**Authors:** Owen R. Jones, Kevin Healy, Julia A. Jones

**Affiliations:** ^1^ Population Biology Group, Department of Biology University of Southern Denmark Odense M Denmark; ^2^ School of Biological Sciences University of Edinburgh Edinburgh UK; ^3^ National University of Ireland, Galway Galway Ireland

## Abstract

Recent comparative analyses have identified positive associations between social organisation and longevity in mammals, but independent replication with larger datasets is needed to establish the robustness of this pattern. Here, we analysed maximum recorded lifespan, body mass, and social organisation data for 1436 mammal species using Bayesian phylogenetic comparative methods, confirming that group‐living and pair‐living species exhibit longer lifespans than solitary species after controlling for body mass and phylogeny. Pair‐living species showed slightly longer lifespans than group‐living species (though credible intervals overlapped), while body mass slopes did not differ substantially among social categories and activity period showed weak associations with lifespan. These results provide independent corroboration of recent findings linking sociality to longevity in mammals and suggest that while group‐living may reduce predation risk, pathogen transmission costs in larger groups may constrain longevity benefits. Our findings, based on the largest comparative dataset analysed to date, strengthen the evidence that social organisation is a key factor shaping mammalian life‐history evolution alongside body size and ecological adaptations.

## Graphical abstract

Using Bayesian phylogenetic analyses of 1436 mammal species, we show that pair‐living and group‐living species have longer maximum lifespans than solitary species after accounting for body mass and phylogeny. Differences between pair‐ and group‐living species were small, and activity period showed weak associations. These findings reinforce social organisation as a key correlate of mammalian longevity.

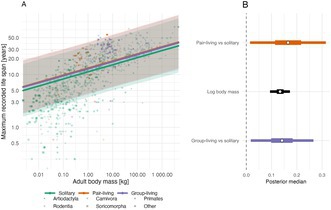

## 1 Introduction

Understanding variation in maximum lifespan among mammals has long engaged evolutionary biologists, as it reflects adaptations shaped by ecological and life‐history factors. Extrinsic mortality, largely driven by predation, imposes strong selective pressures on ageing and longevity (Williams 1957; Reznick et al. 2004). Body size is perhaps the most important factor: larger mammals generally face fewer predators, allowing them to allocate more resources to maintenance and repair, thereby extending their lifespans. In addition to body size, adaptations that reduce predation, such as protective shells, burrowing, flight, or chemical defence, also lower extrinsic mortality, contributing to the evolution of longer lifespans (Healy et al. 2014; Reinke et al. 2022). Comparative analyses of bats and marsupials similarly support reduced environmental vulnerability as a driver of longer lifespan, rather than simple rate‐of‐living predictions (Austad and Fischer 1991). Furthermore, lifespan is correlated with other traits, including age at maturity and parental investment, consistent with the disposable soma theory, which highlights the trade‐off between energy allocation for reproduction and cellular repair. Increasingly, behavioural factors such as sociality are recognised for their impact on lifespan dynamics, adding another dimension to our understanding of longevity evolution (Zhu et al. 2023).

Social groups protect their members from predation and starvation (Alexander 1974; Clutton‐Brock 2002; Wrangham 1980). Reduced risk of death from such extrinsic causes is expected to promote the evolution of longer lifespans (Lucas and Keller 2020; Stearns 1992; Williams and Day 2003). According to evolutionary theories of senescence, a lower rate of extrinsic mortality increases average life expectancy, thereby conferring an adaptive advantage to slow life histories characterised by long lifespans and repeated reproduction (Hamilton 1966; Medawar 1952; Williams 1957). Although this idea has been criticised (Abrams 1993; Moorad et al. 2019), it is broadly supported by empirical data (Gaillard and Lemaître 2017). For instance, among mammals, the ability to fly—an effective strategy to avoid predation—is associated with increased lifespan (Healy et al. 2014). Since group‐living similarly aids predator avoidance, resource defence, and foraging efficiency, we might expect a positive relationship between group‐living and lifespan in comparative analyses; this logic is consistent with selfish‐herd theory, in which individuals reduce their domain of danger by moving towards conspecifics (Hamilton 1971). However a broad‐scale quantitative study of 253 mammalian species failed to detect this relationship (Kamilar et al. 2010), and Lucas and Keller (2020) argued that most rigorous cross‐taxonomic studies have failed to find consistent associations between sociality and longevity. To investigate this unexpected lack of support, we present a re‐analysis of the topic, expanding the sample size to include a greater diversity of mammal species.

To assess whether sociality and longevity correlate, it is first essential to define sociality. Previous studies, such as Kamilar et al. (2010), quantified sociality by examining the median group size for each species and tested whether larger group sizes correlate with lifespan in mammals. However, animal social systems are diverse, ranging from anonymous aggregations to highly cooperative, stable groups of familiar, frequently interacting individuals. These systems also vary widely in group size (from pairs to thousands) and cohesion (from temporary to permanent associations). Each social structure likely represents an evolutionarily stable strategy, adapted to a species' specific ecological and life‐history contexts. This perspective also recognises that group‐living involves trade‐offs, including costs such as food and reproductive competition, increased within‐group conflict, higher risk of disease transmission, and greater visibility to predators. Consequently, while small groups may offer optimal fitness benefits for some species, larger groups may better optimise fitness in others, depending on ecological and predation pressures. This variability raises the question of whether adding an additional group member would have a consistent, additive, linear effect on reducing extrinsic mortality across all mammal species, thereby increasing lifespan. Thus, Kamilar et al.'s (2010) conclusion that sociality does not correlate with longevity in mammals may be premature. To address this question, we re‐examined the relationship using a larger dataset. In this analysis, we coded social systems as a categorical variable with three levels—solitary, pair‐living, and group‐living (Lukas and Clutton‐Brock 2013)—to capture the broader diversity of mammalian social structures and their potential impacts on longevity.

Recently, Zhu et al. (2023) reported a positive association between group‐living and longevity across ~1000 mammal species, using the categorical social organisation framework of Lukas and Clutton‐Brock (2013) and integrating comparative transcriptomics to identify molecular pathways underlying this relationship. Similarly, Salguero‐Gómez (2024) demonstrated across 152 animal species spanning 13 taxonomic classes that more social species live longer, have longer generation times, and postpone maturity compared to solitary species. These studies represent major advances in demonstrating that social organisation predicts longevity when measured appropriately. However, independent replication with different datasets and analytical implementations is essential to establish the robustness of macroevolutionary patterns, particularly given potential sensitivities to phylogenetic trees, lifespan data sources, and species sampling. Here, we provide such an independent analysis using a larger dataset of 1436 mammal species. Although our species set overlaps substantially with that of Zhu et al. (2023)—749 shared species, corresponding to 52.2% of our dataset and 76.9% of theirs (Jaccard similarity = 0.451)—nearly half of our species are unique, providing complementary taxonomic coverage for this independent test.

Lifespan tends to increase with body mass, and larger species generally outlive smaller ones due to a range of interconnected biological and ecological factors, many of which influence extrinsic mortality risk. Perhaps most importantly, larger animals tend to face fewer natural predators, reducing their exposure to extrinsic mortality risk and shifting optimal life‐history strategies towards somatic maintenance to support longer‐term survival. Accompanying these longer‐term prospects is a related shift towards a slower life history, including fewer offspring, delayed reproduction, and extended parental care, aligning with their capacity for repeated reproduction over a longer period. Additionally, larger animals typically have slower metabolic rates, which further contribute to extended lifespans by reducing cellular turnover and oxidative damage. Recognising the importance of extrinsic mortality risk, our analysis controls for body mass to isolate the potential effect of sociality on lifespan, examining whether sociality further enhances longevity beyond what body size alone would predict.

Another trait often assumed to reduce predation risk and, therefore, expected to be associated with long lifespans is nocturnality. Nocturnal species face only predators that can hunt in darkness (Kronfeld‐Schor and Dayan 2003). According to the nocturnal bottleneck hypothesis, early eutherian mammals were strictly nocturnal, avoiding interspecific competition and predation by diurnal reptiles (Gerkema et al. 2013). Reconstructions of primate evolution support the co‐evolution of sociality with a shift in activity pattern from nocturnal to diurnal (Gerkema et al. 2013; Shultz et al. 2011). Sociality may have provided the necessary protection from predation to enable activity during daylight. A previous study found that mammalian lifespans did not differ by activity period (Healy et al. 2014). To check whether Healy et al.'s result rests on an undetected interaction between activity period and sociality, we reanalysed the original data, adding information on social organisation, to ask whether diurnality may be associated with lower lifespans, but only in solitary species.

To shed new light on whether group‐living has led to the evolution of longer lifespans by reducing extrinsic mortality, we analysed data on maximum lifespan, body mass, and sociality for 1436 mammal species. We also added data on the activity period for 611 species. To correct for shared evolutionary history, we used a Bayesian approach following the procedure developed by Healy et al. (2014). Using this approach, we address three predictions. Firstly, we predict that group‐living species will have longer lifespans than pair‐living species, which, in turn, will have longer lifespans than solitary species. Secondly, we expect that body size affects lifespan differently in social and non‐social species. Specifically, we expect non‐social species to gain a greater lifespan benefit from increased body size than social species, which may instead rely on group‐living as an adaptive strategy for predator defence. Finally, we expect diurnal species to generally have shorter lifespans than nocturnal species. However, for diurnal, group‐living species, the risk of predation is likely mitigated through mechanisms associated with group‐living, such as dilution, predator confusion, and collective vigilance (Hamilton 1971; Cresswell and Quinn 2011; Sword et al. 2005), potentially resulting in lifespans comparable to those of nocturnal species of similar body mass.

## 2 Methods

### 2.1 Social Organisation, Activity Period, and Maximum Recorded Lifespan Data

We used three published databases (Healy et al. 2014; Lukas and Clutton‐Brock 2013; Myhrvold et al. 2015), hereafter referred to as the Healy, Lukas, and Myhrvold databases, respectively. The Lukas database, extracted from the appendix of Lukas and Clutton‐Brock (2013), categorises the social organisation of 2454 mammal species. Specifically, it classifies species as solitary, socially monogamous (hereafter, pair‐living; where one adult male and one female share a territory for more than one breeding season), or group‐living. This classification is based on adult home‐range use, with the refinement that cooperatively breeding pairs assisted by non‐breeding offspring are classified as pair‐living rather than group‐living. Because our comparative analysis requires a single species‐level assignment, we retained this published classification rather than attempting ad hoc recoding of species that may vary among populations, seasons, or ecological contexts. Details of data sources and categorisation criteria are provided in Lukas and Clutton‐Brock (2013).

Life history data were sourced from the Myhrvold database, a publicly accessible repository of amniote life history traits (Myhrvold et al. 2015). We extracted two key variables: maximum recorded lifespan (hereafter ‘maximum lifespan’) and adult body mass. Maximum lifespan is regarded as a measure of the pace of life (*sensu* Baudisch 2011) because it is highly correlated with life expectancy from maturity. As an extreme‐value statistic, maximum lifespan is sensitive to population sample size (*N*). Small sample sizes create conservative downward bias in maximum lifespan estimates, meaning poorly‐studied species will tend to have underestimated rather than overestimated lifespans. However, as Finch and Pike (1996) noted, the influence of *N* on maximum lifespan diminishes logarithmically under Gompertz mortality conditions, and maximum lifespan is more influenced by variation in the Gompertz rate parameter than by *N*. Although sexual size dimorphism is common in mammals, our databases typically record the mass of the larger sex. Given the vast range of mass data across species (from 2 g to 4 tons), variation between sexes within a species is minor in comparison. Thus, we are confident in using a single species‐specific measure in our analysis. Thus, we are confident in using a single species‐specific measure in our analysis. The harmonised lifespan data do not consistently preserve the provenance of each longevity record, so we could not distinguish wild from captive estimates in our comparative dataset. Finally, we obtained data on the species' activity period (diurnal, crepuscular, nocturnal or cathemeral) from Healy et al. (2014).

### 2.2 Phylogeny and Taxonomy

We integrated the three datasets described above with a fourth dataset containing phylogenetic information used for phylogenetic correction. Rather than using a single phylogenetic tree, we used an approach developed by Healy et al. (2014) that accounts for the inherent uncertainty in phylogenetic tree topology and dating by using multiple trees, treating them as a Bayesian posterior distribution. Specifically, we used 25 trees sampled from the full set of 101 trees provided by Kuhn et al. (2011), each representing a distinct resolution of polytomies from the ‘best dates’ mammalian supertree phylogeny published by Bininda‐Emonds et al. (2007). We obtained unique species identifiers and updated the trees to reflect the synonymisations recognised by the Integrated Taxonomic Information System (ITIS). We refer to our tree data hereafter as the Kuhn data.

Our taxonomic framework was based on ITIS (www.itis.gov, ITIS 2016), which assigns unique, persistent identifiers, known as Taxonomic Serial Numbers (TSNs), to scientific names. These TSNs were crucial for our analysis. Using the taxize R package (Chamberlain et al. 2020), we retrieved TSNs for each species across the Lukas, Myhrvold, Healy, and Kuhn datasets with the get_tsn function. When the function returned no TSN, we flagged the species, checked for typographical errors, corrected them, and reran get_tsn. For multiple potential matches, we selected the TSN of the valid species. Despite this systematic approach, some species remained unmatched, and we manually reviewed them to identify possible synonyms not listed in ITIS.

Because TSNs may correspond to synonyms rather than valid species names, we used the synonyms function in taxize to retrieve accepted TSNs, thereby consolidating duplicate entries into a single valid species record. In the Lukas dataset, we confirmed that social organisation information remained consistent across merged species. For the Myhrvold dataset, the longest recorded lifespan among merged records was taken as the maximum recorded lifespan. Unused tips were pruned from the phylogenetic trees using the ape package (Paradis and Schliep 2019). This rigorous procedure produced four harmonised datasets, confidently aligned using accepted TSNs. We then filtered the integrated dataset to include only species with complete data on the variables of interest: lifespan and sociality, or lifespan, sociality, and activity period. We also excluded all volant (flying) and gliding mammal species. The final dataset comprised 1436 mammal species from 516 genera and 23 orders, with body masses ranging from 2.33 g (the Shrew species, *Suncus etruscus* , *S. fellowesgordoni* and *S. hosei* ) to 4630 kg (the African Forest Elephant, *Loxodonta cyclotis* ). For analyses involving activity periods, we restricted our sample to 611 species classified as either nocturnal or diurnal in the Healy dataset that matched records in the Lukas dataset.

### 2.3 Statistical Analysis

We examined associations between social organisation, body mass, and maximum recorded lifespan (MRLS) using Bayesian phylogenetic mixed models implemented in the MulTree R package (v1.3.7; Guillerme and Healy 2014), which builds on MCMCglmm (Hadfield 2010). These models account for phylogenetic non‐independence by including a random effect for phylogeny and incorporate phylogenetic uncertainty by fitting each model across multiple plausible trees rather than a single consensus tree.

Before analysis, we log‐transformed (natural logarithm) the maximum recorded lifespan and adult body mass. Body mass was converted from grams to kilogrammes before transformation. Social organisation was modelled as a three‐level factor (solitary, pair‐living, group‐living). For activity analyses, the activity period was represented either as the full factor (diurnal, nocturnal, cathemeral, crepuscular) or, in a focused subset, as a two‐level factor (nocturnal, diurnal).

We fitted a candidate set of models that varied in fixed‐effect structure and data subset (Table 1). The primary inferential models reported in the main text were: (i) an additive sociality model, in which log maximum lifespan was modelled as a function of log body mass and social organisation; (ii) a sociality‐by‐body‐mass interaction model; and (iii) a sociality‐by‐activity interaction model fitted to the nocturnal/diurnal subset. Additional candidate models, including quadratic terms for body mass and alternative activity formulations, were used for sensitivity analyses and are reported in Tables S1–S6.

**TABLE 1:** Candidate model specifications used in the analysis (*n* = 16 models). Models were fitted with mulTree using 25 phylogenetic trees and 4 chains per tree.

Model	Data	Formula
001	Full mammal dataset	Log maximum lifespan ~ log body mass + social organisation
002	Full mammal dataset	Log maximum lifespan ~ log body mass × social organisation
003	Activity‐annotated dataset (all activity categories)	Log maximum lifespan ~ log body mass + social organisation × activity period
004	Activity‐annotated dataset (all activity categories)	Log maximum lifespan ~ log body mass + social organisation + activity period
005	Nocturnal/diurnal subset	Log maximum lifespan ~ log body mass + social organisation × activity period
006	Nocturnal/diurnal subset	Log maximum lifespan ~ log body mass + social organisation + activity period
007	Nocturnal/diurnal subset	Log maximum lifespan ~ log body mass + activity period
008	Nocturnal/diurnal subset	Log maximum lifespan ~ log body mass × activity period
009	Full mammal dataset	Log maximum lifespan ~ log body mass + (log body mass)^2^ + social organisation
010	Full mammal dataset	Log maximum lifespan ~ log body mass + (log body mass)^2^ × social organisation
011	Activity‐annotated dataset (all activity categories)	Log maximum lifespan ~ log body mass + (log body mass)^2^ + social organisation × activity period
012	Activity‐annotated dataset (all activity categories)	Log maximum lifespan ~ log body mass + (log body mass)^2^ + social organisation + activity period
013	Nocturnal/diurnal subset	Log maximum lifespan ~ log body mass + (log body mass)^2^ + social organisation × activity period
014	Nocturnal/diurnal subset	Log maximum lifespan ~ log body mass + (log body mass)^2^ + social organisation + activity period
015	Nocturnal/diurnal subset	Log maximum lifespan ~ log body mass + (log body mass)^2^ + activity period
016	Nocturnal/diurnal subset	Log maximum lifespan ~ log body mass + (log body mass)^2^ × activity period

To assess robustness to reduced sample size, we also refitted the additive sociality model to 30 independent stratified random subsamples retaining 75% of species, with sampling conducted within social‐organisation categories to preserve class balance.

We ran model MCMCs for 12,000 iterations with a burn‐in of 2000 and thinning of 20, using 4 parallel chains. Following Healy et al. (2014), we used inverse‐Wishart priors with *V* = 0.5 and nu = 0.002 for residual and phylogenetic random effects. We evaluated model fit and variance partitioning using DIC (Table S5), marginal and conditional $R^{2}$, and phylogenetic heritability $H^{2}$ (Table S6). We assessed convergence using representative trace plots (Figure S1), Gelman‐Rubin potential scale reduction factors (PSRF; threshold < 1.1; Table S7 and Figure S2), and effective sample size (ESS) diagnostics (Table S8) across all fitted models and trees.

We conducted all analyses in R version 4.5.2 (R Core Team 2026) using the recorded platform shown in the reproducibility output.

## 3 Results

### 3.1 Social Organisation and Lifespan

**FIGURE 1:**
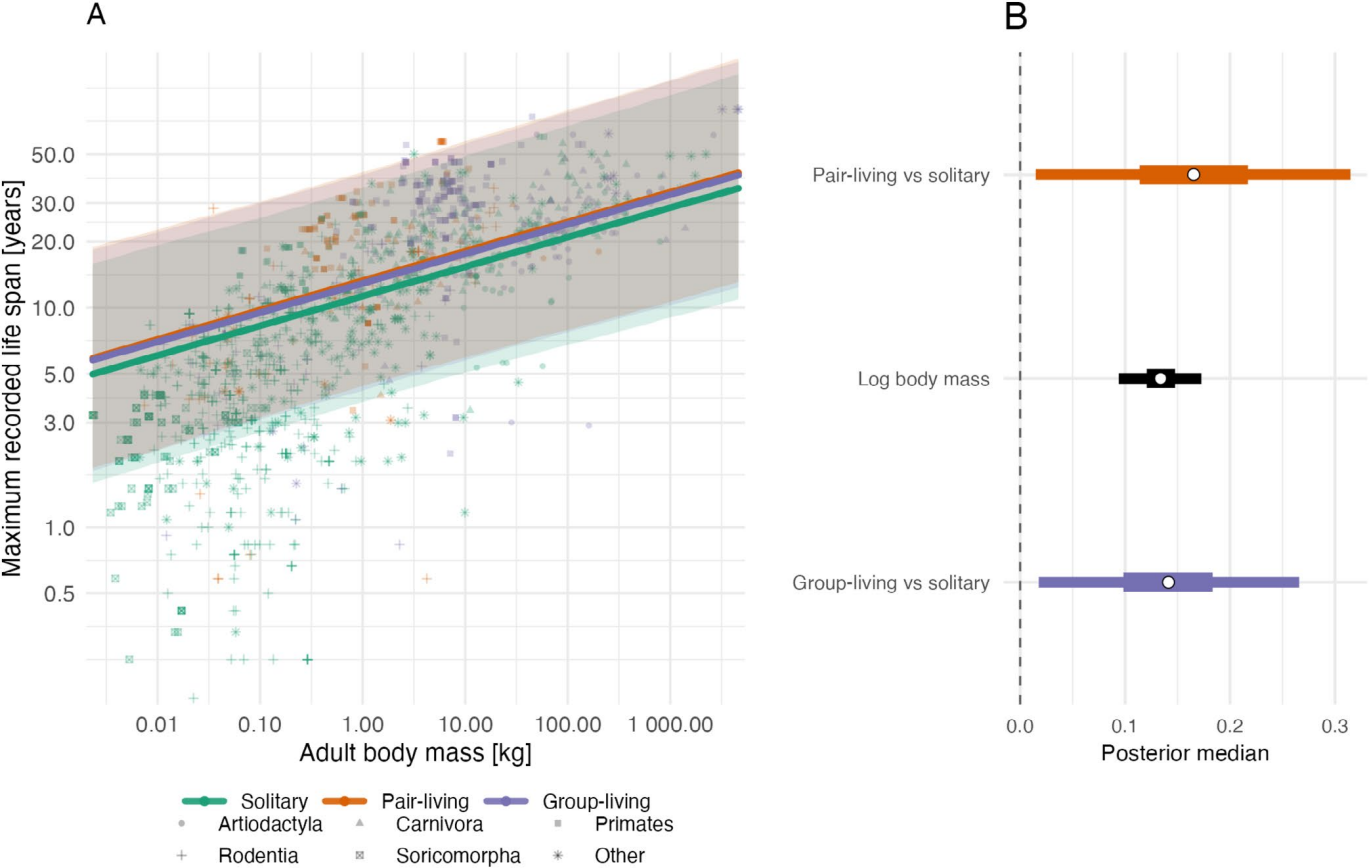
Additive model relating log maximum lifespan to log body mass and social organisation for 1436 mammal species. Panel A shows observed maximum lifespan versus adult body mass (both on log scales): points are species, point colour indicates social organisation (solitary, pair‐living, group‐living), and point shape indicates order (top five orders shown separately; all others grouped as Other). Solid lines are model predictions using posterior median estimates for each social category, with shaded ribbons showing 95% credible intervals. Panel B shows posterior summaries for the fixed effects only: point = median, thick bar = 50% credible interval, thin bar = 95% credible interval. A vertical dashed line marks zero effect.

**FIGURE 2:**
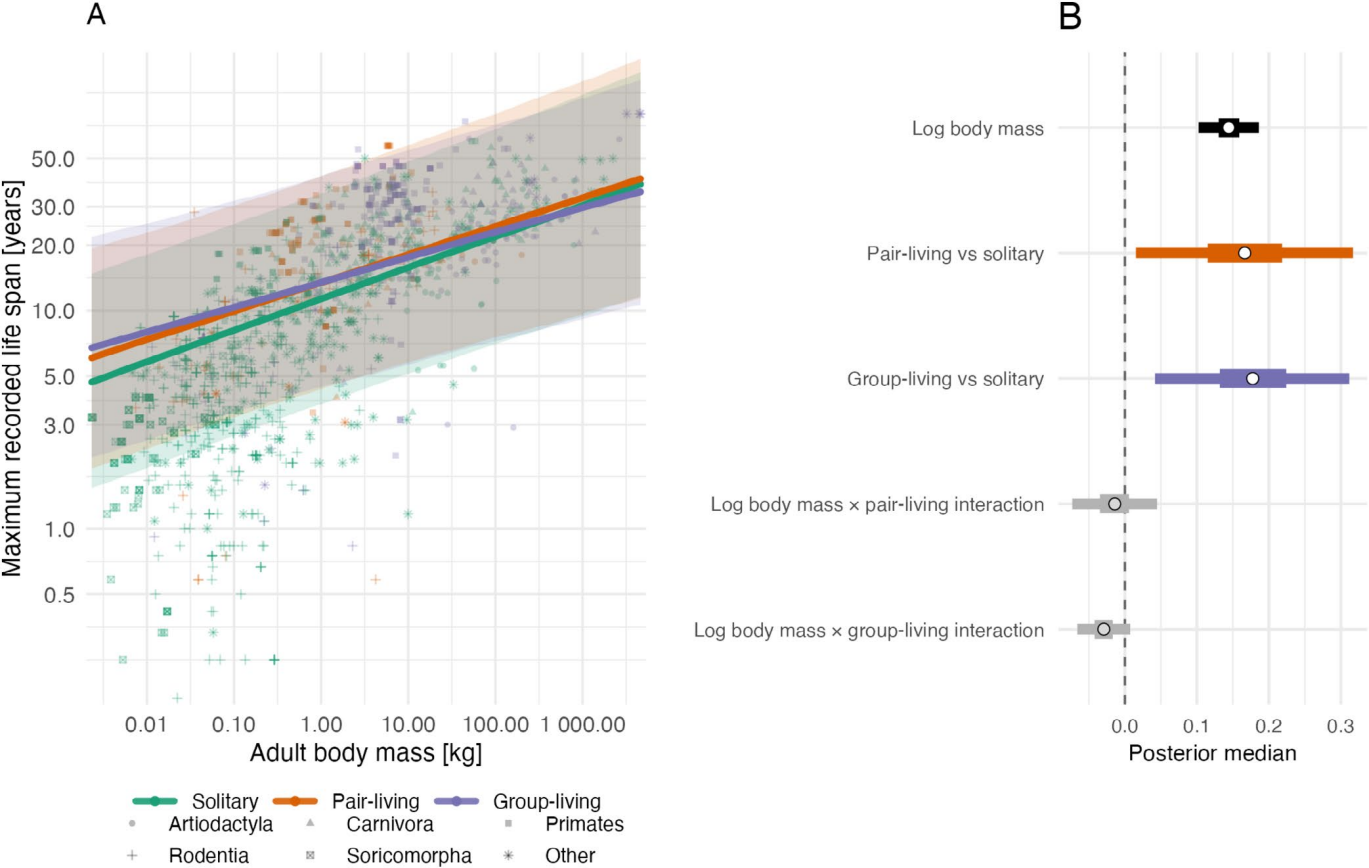
Interaction model relating log maximum lifespan to log body mass, social organisation, and their interaction for 1436 mammal species. Panel A uses the same data display as Figure 1 (log–log axes, colour by social organisation, shape by order), but prediction lines are allowed to differ in both slope and intercept among social categories; shaded ribbons show 95% credible intervals. Panel B presents posterior summaries for the fixed effects only (point = median; thick and thin horizontal bars = 50% and 95% credible intervals, respectively). A vertical dashed line marks zero effect.

In the additive sociality model, pair‐living and group‐living species (‘social species’) had longer lifespans than solitary species at comparable body mass, and pair‐living and group‐living species showed no clear difference (Figure 1; Table 2). In the interaction model, the body‐mass‐by‐sociality interaction terms were weak, and their credible intervals overlapped zero, indicating limited evidence that body‐mass slopes differ strongly among social categories (Figure 2; Table 3). We therefore report both additive and interaction formulations as complementary views of the same relationship.

**TABLE 2:** Additive model relating log maximum lifespan to log adult body mass and social organisation (*n* = 1436). Estimates are posterior modes; intervals are 95% credible intervals (2.5% and 97.5% quantiles).

Coefficient	Estimate	Lower 95% CI	Upper 95% CI
Intercept	2.425	1.300	3.542
Log body mass	0.136	0.094	0.173
Pair‐living vs. solitary	0.166	0.015	0.315
Group‐living vs. solitary	0.139	0.018	0.266
Phylogenetic variance	1.398	1.042	1.814
Residual variance	0.182	0.149	0.217

**TABLE 3:** Interaction model relating log maximum lifespan to log adult body mass, social organisation, and their interaction (*n* = 1436). Estimates are posterior modes with 95% credible intervals.

Coefficient	Estimate	Lower 95% CI	Upper 95% CI
Intercept	2.438	1.316	3.531
Log body mass	0.143	0.102	0.186
Pair‐living vs. solitary	0.167	0.015	0.317
Group‐living vs. solitary	0.178	0.042	0.312
Log body mass × pair‐living interaction	−0.013	−0.073	0.045
Log body mass × group‐living interaction	−0.030	−0.066	0.007
Phylogenetic variance	1.372	1.029	1.797
Residual variance	0.184	0.150	0.218

Quadratic sensitivity analyses (adding a quadratic body‐mass term) did not materially alter the inference about social organisation effects; these model outputs are reported in Tables S1 and S2. For transparency, the complete candidate‐model inventory is given in Table S3 and full coefficient summaries for all fitted models are given in Table S4. Model‐comparison summaries based on DIC are reported in Table S5, and posterior variance‐partition metrics (marginal and conditional $R^{2}$ and phylogenetic $H^{2}$) are reported in Table S6.

A subsampling sensitivity analysis confirmed the robustness of these results: Across 30 independent datasets each containing 75% of species, both pair‐living and group‐living effects remained positive in all replicates. The group‐living effect retained a 95% credible interval excluding zero in 21 of 30 replicates, and the pair‐living effect in 13 of 30 replicates, indicating that the direction of the sociality effect is robust to reduced sampling depth, although precision declines for the weaker pair‐living contrast.

### 3.2 Social Organisation, Activity Period, and Lifespan

**FIGURE 3:**
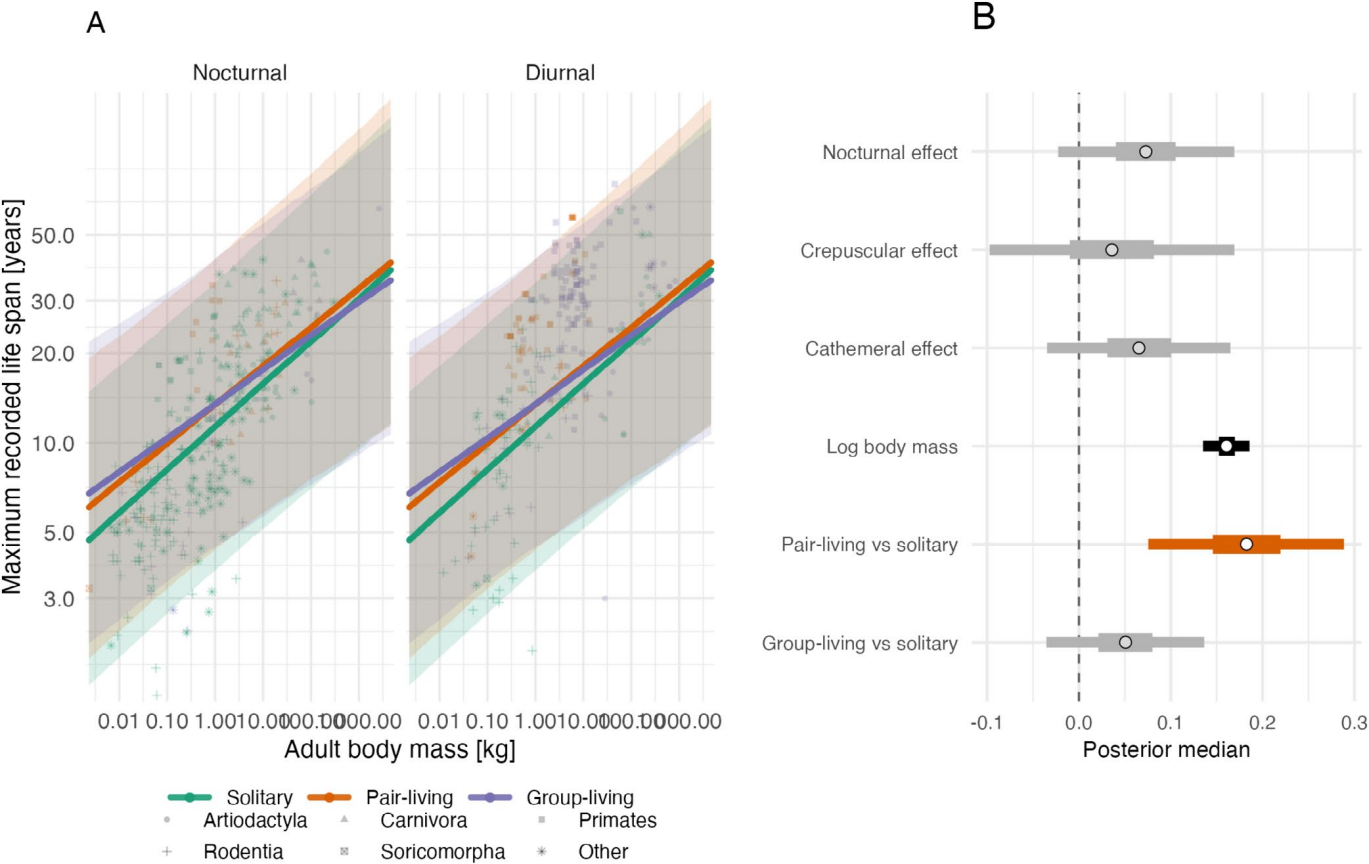
Interaction model relating log maximum lifespan to log body mass, social organisation, activity period, and the social organisation‐by‐activity interaction for the nocturnal/diurnal subset (*n* = 611). Panel A plots observed species values (log–log axes), with colour for social organisation and shape for order; fitted lines are model predictions across the full observed body‐mass range for each social organisation and activity‐period combination, with shaded ribbons showing 95% credible intervals. Panel B gives posterior summaries for the fixed effects only (point = median; thick bar = 50% credible interval; thin bar = 95% credible interval). A vertical dashed line marks zero effect.

In the nocturnal/diurnal subset, evidence for activity‐period effects was weaker than for sociality and body mass (Figure 3; Table 4). Activity main effects and sociality‐by‐activity interaction terms generally had credible intervals that overlapped zero, so these patterns should be interpreted with caution.

**TABLE 4:** Activity‐period interaction model relating log maximum lifespan to log adult body mass, social organisation, activity period, and social organisation‐by‐activity interactions in the nocturnal/diurnal subset (*n* = 611). Estimates are posterior modes with 95% credible intervals.

Coefficient	Estimate	Lower 95% CI	Upper 95% CI
Intercept	2.553	1.915	3.206
Log body mass	0.167	0.139	0.196
Pair‐living vs. solitary	0.125	−0.035	0.286
Group‐living vs. solitary	−0.053	−0.196	0.092
Diurnal vs. nocturnal	−0.100	−0.225	0.023
Pair‐living × diurnality interaction	0.054	−0.174	0.283
Group‐living × diurnality interaction	0.133	−0.056	0.324
Phylogenetic variance	0.430	0.299	0.592
Residual variance	0.061	0.046	0.078

## 4 Discussion

### 4.1 Independent Confirmation of Sociality‐Longevity Associations

Our results provide independent confirmation that social organisation predicts maximum recorded lifespan in mammals, after accounting for body mass and phylogeny. Analysing 1436 species—the largest dataset examined to date—we found that pair‐living and group‐living species both had longer lifespans than solitary species, with no clear difference between the pair‐living and group‐living categories. These findings corroborate the recent transcriptomic and comparative study by Zhu et al. (2023), which reported similar patterns across ~1000 mammalian species using the same categorical social organisation framework as that of Lukas and Clutton‐Brock (2013). The overlap between datasets was substantial but incomplete: 749 species were shared, corresponding to 52.2% of our dataset and 76.9% of Zhu et al.'s dataset (Jaccard similarity = 0.451).

The convergence of results across independent datasets, analytical implementations, and sample compositions strengthens confidence that the sociality‐longevity association is robust and not an artefact of specific methodological choices. While Zhu et al. (2023) found no significant difference between pair‐living and solitary species in their phylogenetic ANOVA, their MCMCglmm results aligned with ours, showing that both pair‐living and group‐living species lived longer than solitary species. Our larger sample size (1436 vs. 974 species) may provide greater power to detect the pair‐living effect, although differences in phylogenetic trees, lifespan data sources, and species composition may also contribute to this subtle divergence. Resolving whether pair‐living consistently confers longevity benefits beyond solitary living will require examining which taxonomic groups and ecological contexts drive this signal. Our findings contrast with the sceptical assessment by Lucas and Keller (2020), who reviewed the literature and concluded that most rigorous cross‐taxonomic studies failed to find consistent associations between sociality and longevity. However, their review predated both our analysis and that of Zhu et al. (2023), and the convergence of results from independent large‐scale comparative studies now provides stronger evidence for a sociality‐longevity association in mammals than was available at the time of their review. The mechanistic evidence from Zhu et al.'s transcriptomics further strengthens this conclusion.

### 4.2 Why Group‐Living Doesn't Dramatically Outlive Pair‐Living: Pathogen Trade‐Offs

A striking finding common to both our study and Zhu et al. (2023) is that group‐living species do not have substantially longer lifespans than pair‐living species, despite theoretical expectations that larger groups provide greater predator dilution, confusion effects, and collective vigilance (Cresswell and Quinn 2011; Sword et al. 2005). This pattern suggests that group size may yield diminishing or even negative marginal returns on longevity beyond the pair‐living state.

One plausible explanation is that pathogen transmission costs rise with group size and density, offsetting the predation benefits of larger groups. Zhu et al.'s (2023) transcriptomic analysis provides molecular support for this trade‐off: they identified immunity‐related genes and pathways that were upregulated in group‐living species, potentially reflecting evolved immune defences against elevated pathogen exposure in denser social aggregations. Social organisation strongly shapes parasite transmission dynamics (Altizer et al. 2003; Kappeler et al. 2015). Group‐living species experience higher contact rates and closer proximity among individuals, creating favourable conditions for infectious disease spread, while also facing altered susceptibility to disease as a result of social competition and stress (Kappeler et al. 2015).

The observation that pair‐living species achieve longevity comparable to that of group‐living species may thus reflect an optimal balance: pairs provide sufficient social benefits—two individuals enable cooperative vigilance, resource defence, and mutual support—without incurring the elevated disease risks of larger groups. This interpretation aligns with life‐history theory emphasising trade‐offs in social evolution (Lucas and Keller 2020) and suggests that the longevity benefits of sociality may saturate or plateau beyond relatively small group sizes, at least across broad comparative scales.

### 4.3 Categorical Social Organisation Reveals Patterns Missed by Group Size

Our findings, together with those of Zhu et al. (2023), demonstrate the value of modelling social organisation as a categorical trait (solitary, pair‐living, group‐living) rather than as a continuous measure of group size. Kamilar et al. (2010) found no relationship between median group size and longevity across 253 mammal species, leading to the conclusion that sociality does not predict longevity. However, both our study and Zhu et al. detect clear associations using the categorical approach pioneered by Lukas and Clutton‐Brock (2013).

This methodological advance likely succeeds because it captures qualitative differences in social structure that continuous group size obscures. The transition from solitary to pair‐living represents a fundamental shift in social organisation—the formation of long‐term social bonds, coordinated activity budgets, and sustained interaction between individuals. Similarly, the transition from pair‐living to group‐living entails new social dynamics, including within‐group competition, dominance hierarchies, and multi‐individual cooperation. These structural differences may have distinct selective consequences for longevity that are not well captured by the number of group members alone. Moreover, group size varies enormously even within the “group‐living” category (from small family groups of 3–5 to herds of hundreds), and this variation likely reflects diverse ecological and demographic pressures that do not uniformly affect longevity.

### 4.4 Body Mass, Activity Period, and the Scope of Social Effects

We found limited evidence that body size affects lifespan differently in social versus solitary species. The body‐mass‐by‐sociality interaction terms in our models were weak, with credible intervals overlapping zero, suggesting that social benefits to longevity operate relatively consistently across the mammalian size spectrum. This contrasts with our prediction that solitary species might gain greater longevity benefits from increased body size (via reduced predation) than social species that rely on group‐based defences. The lack of a clear interaction may indicate that body size and sociality influence longevity through partially independent mechanisms, or that our comparative approach lacks power to detect size‐dependent social effects.

Our analyses of activity period yielded weaker, more uncertain results than those for body mass and social organisation. In the nocturnal/diurnal subset (611 species), activity period main effects and sociality‐by‐activity interactions generally had credible intervals that overlapped zero. This null result is noteworthy given predictions from the nocturnal bottleneck hypothesis (Gerkema et al. 2013) and the idea that diurnal species face higher predation risk without social protection. The absence of clear activity effects suggests either that diurnal and nocturnal predation pressures are more balanced than assumed, or that activity period interacts with sociality and longevity in more complex, context‐dependent ways that our broad comparative models do not capture. Zhu et al. (2023) controlled for activity period in their models but did not test interactions; our focused examination confirms that activity period does not strongly modulate the sociality‐longevity relationship across mammals.

### 4.5 Mechanisms and Macroevolutionary Patterns: Integrating Comparative and Molecular Approaches

While our phylogenetic comparative approach establishes the sociality‐longevity association at the macroevolutionary scale, Zhu et al.'s (2023) transcriptomic analysis provides crucial mechanistic insights. They identified immune‐related and hormonal pathways whose expression correlates with both social organisation and longevity, with particularly striking evidence that group‐living species experience relaxed selection on longevity‐related genes while solitary species show intensified selection. This molecular signature complements our macroevolutionary findings and suggests a feedback loop: social environments enable longer lifespans by reducing extrinsic mortality, which in turn relaxes selection on cellular longevity mechanisms. Future work should test specific mechanistic hypotheses integrating comparative patterns with molecular data—for example, whether immunity gene expression predicts longevity more strongly in group‐living than solitary species, or whether hormonal stress markers mediate social bond quality and individual lifespan within species.

### 4.6 Limitations and Future Directions

Our comparative models identify associations but cannot establish causation. Social organisation, longevity, and other life‐history traits coevolve in response to complex ecological and demographic factors, and distinguishing cause from consequence requires further evidence. Experimental manipulations are rarely feasible at macroevolutionary scales, but within‐species comparisons—examining populations or individuals that vary in sociality—can provide complementary causal insights (Archie et al. 2014; Silk et al. 2010). Similarly, our categorical classification of social organisation, while an improvement over continuous group size, still obscures substantial variation within categories. The “group‐living” category encompasses diverse social systems, from loosely associated or unstable herds to tightly bonded cooperative groups, and these subtypes likely differ in their consequences for longevity. For instance, Thorley (2020) found no consistent evidence that cooperative breeders have longer lifespans than other mammals after controlling for body mass and mode of life, suggesting that specific forms of sociality (e.g., cooperative breeding) may have different longevity consequences than group‐living more generally. Our broad “group‐living” category does not distinguish cooperatively breeding species from other forms of group‐living, or stable from unstable groups, which may obscure important variation.

Maximum recorded lifespan, our response variable, has well‐known limitations as an extreme‐value statistic sensitive to sample size and study effort (Finch and Pike 1996). Because maxima depend on sampling effort, increases in sample size lead to diminishing gains in the recorded maximum, with the largest increases occurring at low sample sizes and progressively smaller gains thereafter; for example, doubling sample size from 50 to 100 individuals is expected to yield a larger increase in observed lifespan than doubling from 100 to 200. As a result, biases associated with sampling effort are expected to be most pronounced for poorly studied species and to decline rapidly as sampling increases, such that moderate differences in sampling effort are unlikely to generate large differences in observed maxima. Variation in study effort among species could therefore influence comparative analyses, particularly where some species are represented by few observations. If social species are better studied than solitary species, this could inflate apparent longevity differences. We also could not distinguish wild from captive longevity records in the harmonised dataset used here; if social species were disproportionately represented by captive records, this could also elevate their apparent maximum lifespan. However, given the broad taxonomic coverage of the dataset and the consistency of results across multiple datasets and analytical approaches, it is unlikely that variation in sampling effort alone explains the observed patterns. This limitation is inherent to large‐scale comparative analyses of longevity and is not unique to the present study. Future comparative work would benefit from improved reporting of per‐species sample sizes and data provenance (e.g., wild versus captive), allowing more explicit evaluation of sampling effects on maximum lifespan estimates. Additionally, finer‐scale analyses within well‐studied clades where social organisation is characterised in greater detail could reveal whether specific social features, such as cooperative breeding, stable bonds, or kin structure, drive longevity effects.

## 5 Conclusions

Our analysis of 1436 mammal species provides robust, independent confirmation that social organisation predicts longevity, even after controlling for body mass and phylogeny. Together with recent molecular evidence (Zhu et al. 2023), these findings confirm that the evolution of sociality and extended lifespan are correlated across the mammalian phylogeny. The pattern appears to reflect reduced extrinsic mortality in social species, although pathogen‐transmission costs may limit the longevity advantages of large groups relative to pairs. These results contribute to a growing understanding of sociality as a key life‐history trait that, alongside body size and ecological specialisations, shapes the extraordinary diversity of ageing and longevity strategies across mammals.

## Author Contributions

**Owen R. Jones:** conceptualisation (equal), data curation (equal), formal analysis (equal), investigation (equal), project administration (equal), software (equal), supervision (equal), validation (equal), visualisation (equal), writing – original draft (equal), writing – review and editing (equal). **Kevin Healy:** methodology (equal), software (equal), validation (equal). **Julia A. Jones:** conceptualisation (equal), data curation (equal), formal analysis (equal), investigation (equal), software (equal), visualisation (equal), writing – original draft (equal).

## Conflicts of Interest

The authors declare no conflicts of interest.

## Supporting information


**Figure S1:** Representative trace plots from the archived MCMC chains for focal parameters in the three primary models. For each model, traces are shown for the parameter most directly tied to the main biological inference and for the phylogenetic random‐effect variance from one representative tree‐level fit. Distinct colours denote the four independent chains. Well‐behaved traces should overlap broadly among chains, fluctuate around a stable mean, and show no persistent trends, abrupt shifts, or long‐term separation, all of which would suggest poor mixing or incomplete convergence.
**Figure S2:** Distribution of worst per‐parameter Gelman‐Rubin potential scale reduction factors (PSRF) across phylogenetic trees for each model. For each tree‐level fit, the plotted value is the largest PSRF among monitored parameters, so each point in the distribution reflects the least well‐mixed parameter for that fit. Values close to 1 indicate good convergence among chains, whereas values approaching or exceeding the dashed 1.1 threshold indicate that at least one parameter may require more sampling.
**Table S1:** Posterior summaries for Model 9, the quadratic additive sociality model (log maximum lifespan ~ log body mass + (log body mass)2 + social organisation; *n* = 1436). Estimates are posterior modes with 95% credible intervals. Coefficients whose intervals exclude zero have strongest support for directional effects on log maximum lifespan.
**Table S2:** Posterior summaries for Model 10, the quadratic sociality‐by‐body‐mass interaction model (log maximum lifespan ~ log body mass + (log body mass)2 × social organisation; *n* = 1436). Estimates are posterior modes with 95% credible intervals. Interaction terms test whether linear and quadratic body‐mass effects differ among social‐organisation categories.
**Table S3:** Complete candidate model inventory. For each model, we report the model identifier, analysis dataset, number of species, and fixed‐effects specification. This table defines the full model set used for primary and sensitivity analyses.
**Table S4:** Posterior summaries for all coefficients across the full candidate model set (Models 1–16). Estimates are posterior modes with 95% credible intervals. Term type distinguishes fixed effects from variance components, enabling comparison of parameter support across models.
**Table S5:** Deviance information criterion (DIC) across archived tree‐by‐chain fits for each model. For each candidate model, the table reports the number of archived fits contributing to the summary, together with the median, 2.5% quantile, and 97.5% quantile of DIC across phylogenetic‐tree replicates and MCMC chains. Lower DIC indicates a better trade‐off between model fit and effective complexity, so these values provide a relative comparison among the archived candidate models rather than an absolute measure of fit.
**Table S6:** Posterior summaries of variance partitioning for each model. Marginal R2 is the proportion of total variance explained by fixed effects alone, conditional R2 is the proportion explained by fixed effects plus the phylogenetic random effect, and phylogenetic heritability (H2) is the proportion of non‐fixed‐effect variance attributable to phylogeny. Entries report posterior medians with 95% credible intervals pooled across archived tree‐by‐chain fits, allowing comparison of how much model variation is associated with fixed predictors versus phylogenetic structure.
**Table S7:** Gelman‐Rubin convergence diagnostics (PSRF) by model across tree‐level fits. Reported values include the median and maximum multivariate PSRF (mPSRF), together with the median and worst per‐parameter PSRF among monitored parameters. Values close to 1 indicate good agreement among chains, whereas persistent values above approximately 1.1 suggest that at least some parameters may not have mixed fully.
**Table S8:** Effective sample size (ESS) diagnostics by model across all archived chain files. For each chain, ESS was calculated for all fixed and variance parameters, and the summary is based on the minimum ESS within that chain (i.e., the worst‐sampled parameter). Higher ESS indicates greater Monte Carlo precision, whereas counts below 200 and 500 provide conservative warning thresholds for potentially under‐sampled chains.

## Data Availability

All data and code required to reproduce this study are archived on Zenodo: https://doi.org/10.5281/zenodo.18665209.
