# Supplementary material for: Social Organisation Predicts Lifespan in Mammals
Source: Ecol Evol. 2026 Apr 28;16(5):e73587. doi: 10.1002/ece3.73587 (PMC13122425; doi:10.1002/ece3.73587)
Supplement: Supplementary file 1 — Figure S1: Representative trace plots from the archived MCMC chains for focal parameters in the three primary models. For each model, traces are shown for the parameter most directly tied to the main biological inference and for the phylogenetic random‐effect variance from one representative tree‐level fit. Distinct colours denote the four independent chains. Well‐behaved traces should overlap broadly among chains, fluctuate around a stable mean, and show no persistent trends, abrupt shifts, or long‐term separation, all of which would suggest poor mixing or incomplete convergence. Figure S2: Distribution of worst per‐parameter Gelman‐Rubin potential scale reduction factors (PSRF) across phylogenetic trees for each model. For each tree‐level fit, the plotted value is the largest PSRF among monitored parameters, so each point in the distribution reflects the least well‐mixed parameter for that fit. Values close to 1 indicate good convergence among chains, whereas values approaching or exceeding the dashed 1.1 threshold indicate that at least one parameter may require more sampling. Table S1: Posterior summaries for Model 9, the quadratic additive sociality model (log maximum lifespan ~ log body mass + (log body mass)2 + social organisation; n = 1436). Estimates are posterior modes with 95% credible intervals. Coefficients whose intervals exclude zero have strongest support for directional effects on log maximum lifespan. Table S2: Posterior summaries for Model 10, the quadratic sociality‐by‐body‐mass interaction model (log maximum lifespan ~ log body mass + (log body mass)2 × social organisation; n = 1436). Estimates are posterior modes with 95% credible intervals. Interaction terms test whether linear and quadratic body‐mass effects differ among social‐organisation categories. Table S3: Complete candidate model inventory. For each model, we report the model identifier, analysis dataset, number of species, and fixed‐effects specification. This table defines the full m [file ECE3-16-e73587-s001.docx]

Supporting Information: Social organisation predicts lifespan in mammals

# Supporting Information

The supplementary tables below document the full model set and diagnostics. In addition to the focal models shown in the main text, we provide complete candidate-model specifications and posterior summaries for all 16 fitted candidate models. Abbreviations used here follow the main text: MRLS = maximum recorded lifespan, PSRF = potential scale reduction factor, and ESS = effective sample size.

## Sensitivity analyses (Tables S1-S2)

Table S1. Posterior summaries for Model 9, the quadratic additive sociality model (log maximum lifespan ~ log body mass + (log body mass)^2 + social organisation; n = 1,436). Estimates are posterior modes with 95% credible intervals. Coefficients whose intervals exclude zero have strongest support for directional effects on log maximum lifespan.

| Coefficient | Estimate | Lower 95% CI | Upper 95% CI |
| --- | --- | --- | --- |
| Intercept | 2.427 | 1.309 | 3.517 |
| Log body mass | 0.133 | 0.093 | 0.173 |
| (Log body mass)^2 | 0.001 | -0.006 | 0.008 |
| Pair-living vs solitary | 0.166 | 0.014 | 0.315 |
| Group-living vs solitary | 0.140 | 0.014 | 0.264 |
| phylogenetic.variance | 1.380 | 1.045 | 1.815 |
| residual.variance | 0.183 | 0.150 | 0.217 |

Table S2. Posterior summaries for Model 10, the quadratic sociality-by-body-mass interaction model (log maximum lifespan ~ log body mass + (log body mass)^2 × social organisation; n = 1,436). Estimates are posterior modes with 95% credible intervals. Interaction terms test whether linear and quadratic body-mass effects differ among social-organisation categories.

| Coefficient | Estimate | Lower 95% CI | Upper 95% CI |
| --- | --- | --- | --- |
| Intercept | 2.438 | 1.292 | 3.525 |
| Log body mass | 0.134 | 0.093 | 0.175 |
| (Log body mass)^2 | 0.002 | -0.006 | 0.010 |
| Pair-living vs solitary | 0.185 | -0.005 | 0.384 |
| Group-living vs solitary | 0.165 | 0.000 | 0.333 |
| (Log body mass)^2 × pair-living interaction | -0.004 | -0.023 | 0.016 |
| (Log body mass)^2 × group-living interaction | -0.003 | -0.012 | 0.007 |
| phylogenetic.variance | 1.394 | 1.055 | 1.809 |
| residual.variance | 0.183 | 0.150 | 0.217 |

## Full candidate model set (Tables S3-S4)

Table S3. Complete candidate model inventory. For each model, we report the model identifier, analysis dataset, number of species, and fixed-effects specification. This table defines the full model set used for primary and sensitivity analyses.

| Model | Dataset | Species (n) | Model specification |
| --- | --- | --- | --- |
| 001 | Full mammal dataset | 1,436 | Log maximum lifespan ~ log body mass + social organisation |
| 002 | Full mammal dataset | 1,436 | Log maximum lifespan ~ log body mass × social organisation |
| 003 | Activity-annotated dataset (all activity categories) | 804 | Log maximum lifespan ~ log body mass + social organisation × activity period |
| 004 | Activity-annotated dataset (all activity categories) | 804 | Log maximum lifespan ~ log body mass + social organisation + activity period |
| 005 | Nocturnal/diurnal subset | 611 | Log maximum lifespan ~ log body mass + social organisation × activity period |
| 006 | Nocturnal/diurnal subset | 611 | Log maximum lifespan ~ log body mass + social organisation + activity period |
| 007 | Nocturnal/diurnal subset | 611 | Log maximum lifespan ~ log body mass + activity period |
| 008 | Nocturnal/diurnal subset | 611 | Log maximum lifespan ~ log body mass × activity period |
| 009 | Full mammal dataset | 1,436 | Log maximum lifespan ~ log body mass + (log body mass)^2 + social organisation |
| 010 | Full mammal dataset | 1,436 | Log maximum lifespan ~ log body mass + (log body mass)^2 × social organisation |
| 011 | Activity-annotated dataset (all activity categories) | 804 | Log maximum lifespan ~ log body mass + (log body mass)^2 + social organisation × activity period |
| 012 | Activity-annotated dataset (all activity categories) | 804 | Log maximum lifespan ~ log body mass + (log body mass)^2 + social organisation + activity period |
| 013 | Nocturnal/diurnal subset | 611 | Log maximum lifespan ~ log body mass + (log body mass)^2 + social organisation × activity period |
| 014 | Nocturnal/diurnal subset | 611 | Log maximum lifespan ~ log body mass + (log body mass)^2 + social organisation + activity period |
| 015 | Nocturnal/diurnal subset | 611 | Log maximum lifespan ~ log body mass + (log body mass)^2 + activity period |
| 016 | Nocturnal/diurnal subset | 611 | Log maximum lifespan ~ log body mass + (log body mass)^2 × activity period |

Table S4. Posterior summaries for all coefficients across the full candidate model set (Models 1-16). Estimates are posterior modes with 95% credible intervals. Term type distinguishes fixed effects from variance components, enabling comparison of parameter support across models.

| Model | Term type | Coefficient | Estimate | Lower 95% CI | Upper 95% CI |
| --- | --- | --- | --- | --- | --- |
| 001 | Fixed effect | Intercept | 2.425 | 1.300 | 3.542 |
| 001 | Fixed effect | Log body mass | 0.136 | 0.094 | 0.173 |
| 001 | Fixed effect | Pair-living vs solitary | 0.166 | 0.015 | 0.315 |
| 001 | Fixed effect | Group-living vs solitary | 0.139 | 0.018 | 0.266 |
| 001 | Variance component | phylogenetic.variance | 1.398 | 1.042 | 1.814 |
| 001 | Variance component | residual.variance | 0.182 | 0.149 | 0.217 |
| 002 | Fixed effect | Intercept | 2.438 | 1.316 | 3.531 |
| 002 | Fixed effect | Log body mass | 0.143 | 0.102 | 0.186 |
| 002 | Fixed effect | Pair-living vs solitary | 0.167 | 0.015 | 0.317 |
| 002 | Fixed effect | Group-living vs solitary | 0.178 | 0.042 | 0.312 |
| 002 | Fixed effect | Log body mass × pair-living interaction | -0.014 | -0.073 | 0.045 |
| 002 | Fixed effect | Log body mass × group-living interaction | -0.030 | -0.066 | 0.007 |
| 002 | Variance component | phylogenetic.variance | 1.372 | 1.029 | 1.797 |
| 002 | Variance component | residual.variance | 0.184 | 0.150 | 0.218 |
| 003 | Fixed effect | Intercept | 2.490 | 1.873 | 3.104 |
| 003 | Fixed effect | Log body mass | 0.161 | 0.136 | 0.186 |
| 003 | Fixed effect | Pair-living vs solitary | 0.202 | 0.024 | 0.378 |
| 003 | Fixed effect | Group-living vs solitary | 0.110 | -0.019 | 0.240 |
| 003 | Fixed effect | activityPeriodB.nocturnal | 0.119 | -0.003 | 0.237 |
| 003 | Fixed effect | activityPeriodC.cathemeral | 0.070 | -0.062 | 0.205 |
| 003 | Fixed effect | activityPeriodD.crepuscular | 0.107 | -0.080 | 0.274 |
| 003 | Fixed effect | socialOrgB.PairLiving:activityPeriodB.nocturnal | -0.051 | -0.275 | 0.180 |
| 003 | Fixed effect | socialOrgC.GroupLiving:activityPeriodB.nocturnal | -0.138 | -0.333 | 0.044 |
| 003 | Fixed effect | socialOrgB.PairLiving:activityPeriodC.cathemeral | 0.053 | -0.192 | 0.302 |
| 003 | Fixed effect | socialOrgC.GroupLiving:activityPeriodC.cathemeral | -0.011 | -0.208 | 0.185 |
| 003 | Fixed effect | socialOrgB.PairLiving:activityPeriodD.crepuscular | -0.060 | -0.468 | 0.331 |
| 003 | Fixed effect | socialOrgC.GroupLiving:activityPeriodD.crepuscular | -0.153 | -0.435 | 0.123 |
| 003 | Variance component | phylogenetic.variance | 0.404 | 0.287 | 0.545 |
| 003 | Variance component | residual.variance | 0.068 | 0.053 | 0.083 |
| 004 | Fixed effect | Intercept | 2.524 | 1.918 | 3.141 |
| 004 | Fixed effect | Log body mass | 0.160 | 0.135 | 0.186 |
| 004 | Fixed effect | Pair-living vs solitary | 0.187 | 0.075 | 0.289 |
| 004 | Fixed effect | Group-living vs solitary | 0.051 | -0.035 | 0.136 |
| 004 | Fixed effect | activityPeriodB.nocturnal | 0.073 | -0.023 | 0.169 |
| 004 | Fixed effect | activityPeriodC.cathemeral | 0.066 | -0.035 | 0.165 |
| 004 | Fixed effect | activityPeriodD.crepuscular | 0.036 | -0.097 | 0.169 |
| 004 | Variance component | phylogenetic.variance | 0.409 | 0.294 | 0.548 |
| 004 | Variance component | residual.variance | 0.066 | 0.053 | 0.082 |
| 005 | Fixed effect | Intercept | 2.553 | 1.915 | 3.206 |
| 005 | Fixed effect | Log body mass | 0.167 | 0.139 | 0.196 |
| 005 | Fixed effect | Pair-living vs solitary | 0.125 | -0.035 | 0.286 |
| 005 | Fixed effect | Group-living vs solitary | -0.053 | -0.196 | 0.092 |
| 005 | Fixed effect | Diurnality effect | -0.100 | -0.225 | 0.023 |
| 005 | Fixed effect | Pair-living × diurnality interaction | 0.054 | -0.174 | 0.283 |
| 005 | Fixed effect | Group-living × diurnality interaction | 0.133 | -0.056 | 0.324 |
| 005 | Variance component | phylogenetic.variance | 0.430 | 0.299 | 0.592 |
| 005 | Variance component | residual.variance | 0.061 | 0.046 | 0.078 |
| 006 | Fixed effect | Intercept | 2.540 | 1.905 | 3.220 |
| 006 | Fixed effect | Log body mass | 0.168 | 0.139 | 0.196 |
| 006 | Fixed effect | Pair-living vs solitary | 0.138 | 0.014 | 0.260 |
| 006 | Fixed effect | Group-living vs solitary | 0.021 | -0.079 | 0.119 |
| 006 | Fixed effect | Diurnality effect | -0.057 | -0.162 | 0.046 |
| 006 | Variance component | phylogenetic.variance | 0.443 | 0.306 | 0.607 |
| 006 | Variance component | residual.variance | 0.060 | 0.045 | 0.077 |
| 007 | Fixed effect | Intercept | 2.561 | 1.902 | 3.232 |
| 007 | Fixed effect | Log body mass | 0.165 | 0.138 | 0.195 |
| 007 | Fixed effect | Diurnality effect | -0.053 | -0.155 | 0.052 |
| 007 | Variance component | phylogenetic.variance | 0.453 | 0.324 | 0.616 |
| 007 | Variance component | residual.variance | 0.059 | 0.044 | 0.076 |
| 008 | Fixed effect | Intercept | 2.573 | 1.911 | 3.231 |
| 008 | Fixed effect | Log body mass | 0.162 | 0.130 | 0.192 |
| 008 | Fixed effect | Diurnality effect | -0.065 | -0.173 | 0.041 |
| 008 | Fixed effect | Log body mass × diurnality interaction | 0.018 | -0.017 | 0.051 |
| 008 | Variance component | phylogenetic.variance | 0.456 | 0.318 | 0.618 |
| 008 | Variance component | residual.variance | 0.059 | 0.044 | 0.076 |
| 009 | Fixed effect | Intercept | 2.427 | 1.309 | 3.517 |
| 009 | Fixed effect | Log body mass | 0.133 | 0.093 | 0.173 |
| 009 | Fixed effect | (Log body mass)^2 | 0.001 | -0.006 | 0.008 |
| 009 | Fixed effect | Pair-living vs solitary | 0.166 | 0.014 | 0.315 |
| 009 | Fixed effect | Group-living vs solitary | 0.140 | 0.014 | 0.264 |
| 009 | Variance component | phylogenetic.variance | 1.380 | 1.045 | 1.815 |
| 009 | Variance component | residual.variance | 0.183 | 0.150 | 0.217 |
| 010 | Fixed effect | Intercept | 2.438 | 1.292 | 3.525 |
| 010 | Fixed effect | Log body mass | 0.134 | 0.093 | 0.175 |
| 010 | Fixed effect | (Log body mass)^2 | 0.002 | -0.006 | 0.010 |
| 010 | Fixed effect | Pair-living vs solitary | 0.185 | -0.005 | 0.384 |
| 010 | Fixed effect | Group-living vs solitary | 0.165 | 0.000 | 0.333 |
| 010 | Fixed effect | (Log body mass)^2 × pair-living interaction | -0.004 | -0.023 | 0.016 |
| 010 | Fixed effect | (Log body mass)^2 × group-living interaction | -0.003 | -0.012 | 0.007 |
| 010 | Variance component | phylogenetic.variance | 1.394 | 1.055 | 1.809 |
| 010 | Variance component | residual.variance | 0.183 | 0.150 | 0.217 |
| 011 | Fixed effect | Intercept | 2.481 | 1.871 | 3.097 |
| 011 | Fixed effect | Log body mass | 0.160 | 0.134 | 0.186 |
| 011 | Fixed effect | (Log body mass)^2 | 0.001 | -0.004 | 0.006 |
| 011 | Fixed effect | Pair-living vs solitary | 0.202 | 0.025 | 0.380 |
| 011 | Fixed effect | Group-living vs solitary | 0.108 | -0.021 | 0.240 |
| 011 | Fixed effect | activityPeriodB.nocturnal | 0.121 | -0.002 | 0.237 |
| 011 | Fixed effect | activityPeriodC.cathemeral | 0.072 | -0.060 | 0.205 |
| 011 | Fixed effect | activityPeriodD.crepuscular | 0.097 | -0.082 | 0.272 |
| 011 | Fixed effect | socialOrgB.PairLiving:activityPeriodB.nocturnal | -0.046 | -0.277 | 0.180 |
| 011 | Fixed effect | socialOrgC.GroupLiving:activityPeriodB.nocturnal | -0.138 | -0.333 | 0.044 |
| 011 | Fixed effect | socialOrgB.PairLiving:activityPeriodC.cathemeral | 0.051 | -0.199 | 0.303 |
| 011 | Fixed effect | socialOrgC.GroupLiving:activityPeriodC.cathemeral | -0.015 | -0.210 | 0.187 |
| 011 | Fixed effect | socialOrgB.PairLiving:activityPeriodD.crepuscular | -0.063 | -0.474 | 0.336 |
| 011 | Fixed effect | socialOrgC.GroupLiving:activityPeriodD.crepuscular | -0.150 | -0.440 | 0.120 |
| 011 | Variance component | phylogenetic.variance | 0.405 | 0.291 | 0.543 |
| 011 | Variance component | residual.variance | 0.068 | 0.054 | 0.083 |
| 012 | Fixed effect | Intercept | 2.513 | 1.914 | 3.128 |
| 012 | Fixed effect | Log body mass | 0.159 | 0.133 | 0.186 |
| 012 | Fixed effect | (Log body mass)^2 | 0.001 | -0.004 | 0.006 |
| 012 | Fixed effect | Pair-living vs solitary | 0.174 | 0.074 | 0.288 |
| 012 | Fixed effect | Group-living vs solitary | 0.049 | -0.037 | 0.135 |
| 012 | Fixed effect | activityPeriodB.nocturnal | 0.072 | -0.023 | 0.168 |
| 012 | Fixed effect | activityPeriodC.cathemeral | 0.061 | -0.034 | 0.166 |
| 012 | Fixed effect | activityPeriodD.crepuscular | 0.031 | -0.098 | 0.168 |
| 012 | Variance component | phylogenetic.variance | 0.403 | 0.294 | 0.550 |
| 012 | Variance component | residual.variance | 0.066 | 0.053 | 0.082 |
| 013 | Fixed effect | Intercept | 2.566 | 1.909 | 3.213 |
| 013 | Fixed effect | Log body mass | 0.166 | 0.138 | 0.196 |
| 013 | Fixed effect | (Log body mass)^2 | 0.000 | -0.005 | 0.007 |
| 013 | Fixed effect | Pair-living vs solitary | 0.125 | -0.037 | 0.286 |
| 013 | Fixed effect | Group-living vs solitary | -0.056 | -0.201 | 0.091 |
| 013 | Fixed effect | Diurnality effect | -0.104 | -0.228 | 0.023 |
| 013 | Fixed effect | Pair-living × diurnality interaction | 0.058 | -0.174 | 0.286 |
| 013 | Fixed effect | Group-living × diurnality interaction | 0.140 | -0.055 | 0.326 |
| 013 | Variance component | phylogenetic.variance | 0.434 | 0.298 | 0.594 |
| 013 | Variance component | residual.variance | 0.061 | 0.046 | 0.078 |
| 014 | Fixed effect | Intercept | 2.552 | 1.899 | 3.213 |
| 014 | Fixed effect | Log body mass | 0.169 | 0.138 | 0.196 |
| 014 | Fixed effect | (Log body mass)^2 | 0.000 | -0.006 | 0.006 |
| 014 | Fixed effect | Pair-living vs solitary | 0.138 | 0.013 | 0.260 |
| 014 | Fixed effect | Group-living vs solitary | 0.020 | -0.080 | 0.119 |
| 014 | Fixed effect | Diurnality effect | -0.057 | -0.162 | 0.048 |
| 014 | Variance component | phylogenetic.variance | 0.446 | 0.310 | 0.607 |
| 014 | Variance component | residual.variance | 0.059 | 0.045 | 0.077 |
| 015 | Fixed effect | Intercept | 2.582 | 1.901 | 3.230 |
| 015 | Fixed effect | Log body mass | 0.166 | 0.137 | 0.195 |
| 015 | Fixed effect | (Log body mass)^2 | 0.001 | -0.006 | 0.006 |
| 015 | Fixed effect | Diurnality effect | -0.052 | -0.157 | 0.052 |
| 015 | Variance component | phylogenetic.variance | 0.458 | 0.324 | 0.623 |
| 015 | Variance component | residual.variance | 0.059 | 0.044 | 0.075 |
| 016 | Fixed effect | Intercept | 2.562 | 1.879 | 3.230 |
| 016 | Fixed effect | Log body mass | 0.169 | 0.139 | 0.198 |
| 016 | Fixed effect | (Log body mass)^2 | 0.002 | -0.005 | 0.009 |
| 016 | Fixed effect | Diurnality effect | -0.005 | -0.139 | 0.125 |
| 016 | Fixed effect | (Log body mass)^2 × diurnality interaction | -0.005 | -0.015 | 0.004 |
| 016 | Variance component | phylogenetic.variance | 0.458 | 0.328 | 0.628 |
| 016 | Variance component | residual.variance | 0.058 | 0.044 | 0.075 |

## Model fit and variance partitioning (Tables S5-S6)

Pointwise log-likelihoods were not retained in the archived MCMCglmm chain objects, so leave-one-out cross-validation (LOO) and the widely applicable information criterion (WAIC) could not be calculated retrospectively from the saved outputs alone. As the available model-comparison statistic, we therefore report the deviance information criterion (DIC) across the archived chain-by-tree fits. We also summarise posterior variance partitioning for each model using marginal $R^{2}$ (variance explained by fixed effects), conditional $R^{2}$ (variance explained by fixed plus phylogenetic random effects), and phylogenetic heritability $H^{2}$ (the proportion of non-fixed-effect variance attributable to phylogeny).

Table S5. Deviance information criterion (DIC) across archived tree-by-chain fits for each model. For each candidate model, the table reports the number of archived fits contributing to the summary, together with the median, 2.5% quantile, and 97.5% quantile of DIC across phylogenetic-tree replicates and MCMC chains. Lower DIC indicates a better trade-off between model fit and effective complexity, so these values provide a relative comparison among the archived candidate models rather than an absolute measure of fit.

| Model | Archived fits | Median DIC | Lower 95% quantile | Upper 95% quantile |
| --- | --- | --- | --- | --- |
| 001 | 200 | 2,170.36 | 2,025.21 | 2,247.33 |
| 002 | 200 | 2,174.56 | 2,033.73 | 2,250.74 |
| 003 | 100 | 438.88 | 385.91 | 465.06 |
| 004 | 100 | 426.05 | 377.17 | 461.84 |
| 005 | 200 | 284.72 | 249.00 | 313.56 |
| 006 | 100 | 277.79 | 239.61 | 307.56 |
| 007 | 100 | 273.04 | 234.46 | 304.40 |
| 008 | 100 | 275.42 | 233.89 | 309.08 |
| 009 | 100 | 2,173.56 | 2,028.22 | 2,248.21 |
| 010 | 100 | 2,173.65 | 2,036.82 | 2,250.14 |
| 011 | 100 | 439.40 | 387.60 | 463.42 |
| 012 | 100 | 428.59 | 374.85 | 456.64 |
| 013 | 100 | 285.61 | 254.97 | 316.65 |
| 014 | 100 | 278.09 | 241.94 | 311.20 |
| 015 | 100 | 274.89 | 233.60 | 305.96 |
| 016 | 100 | 270.86 | 227.57 | 297.41 |
| 0NA | 3,012 | 1,719.61 | 1,456.70 | 1,860.63 |

Table S6. Posterior summaries of variance partitioning for each model. Marginal R² is the proportion of total variance explained by fixed effects alone, conditional R² is the proportion explained by fixed effects plus the phylogenetic random effect, and phylogenetic heritability (H²) is the proportion of non-fixed-effect variance attributable to phylogeny. Entries report posterior medians with 95% credible intervals pooled across archived tree-by-chain fits, allowing comparison of how much model variation is associated with fixed predictors versus phylogenetic structure.

| Model | Marginal R² | Conditional R² | Phylogenetic H² |
| --- | --- | --- | --- |
| 001 | 0.102 [0.053, 0.164] | 0.897 [0.857, 0.930] | 0.885 [0.835, 0.924] |
| 002 | 0.107 [0.057, 0.171] | 0.896 [0.855, 0.929] | 0.883 [0.832, 0.922] |
| 003 | 0.321 [0.230, 0.415] | 0.903 [0.867, 0.932] | 0.858 [0.786, 0.908] |
| 004 | 0.313 [0.224, 0.406] | 0.904 [0.869, 0.933] | 0.860 [0.792, 0.909] |
| 005 | 0.276 [0.188, 0.373] | 0.911 [0.870, 0.942] | 0.877 [0.805, 0.925] |
| 006 | 0.268 [0.181, 0.365] | 0.913 [0.872, 0.944] | 0.882 [0.809, 0.928] |
| 007 | 0.258 [0.174, 0.350] | 0.915 [0.876, 0.945] | 0.885 [0.820, 0.930] |
| 008 | 0.261 [0.177, 0.355] | 0.916 [0.875, 0.945] | 0.886 [0.817, 0.930] |
| 009 | 0.102 [0.053, 0.163] | 0.897 [0.857, 0.930] | 0.885 [0.836, 0.924] |
| 010 | 0.103 [0.054, 0.166] | 0.897 [0.858, 0.929] | 0.885 [0.836, 0.923] |
| 011 | 0.320 [0.231, 0.413] | 0.904 [0.869, 0.932] | 0.858 [0.790, 0.907] |
| 012 | 0.312 [0.223, 0.406] | 0.904 [0.869, 0.934] | 0.860 [0.793, 0.910] |
| 013 | 0.277 [0.188, 0.374] | 0.911 [0.870, 0.942] | 0.877 [0.803, 0.925] |
| 014 | 0.268 [0.182, 0.363] | 0.914 [0.873, 0.944] | 0.882 [0.811, 0.928] |
| 015 | 0.258 [0.174, 0.350] | 0.916 [0.877, 0.945] | 0.886 [0.821, 0.931] |
| 016 | 0.256 [0.173, 0.348] | 0.917 [0.878, 0.946] | 0.888 [0.823, 0.932] |

## Convergence diagnostics (Tables S7-S8; Figures S1-S2)


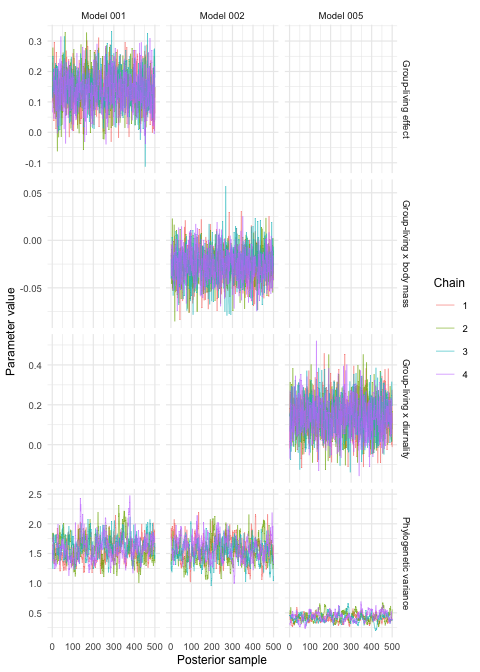


Figure S1. Representative trace plots from the archived MCMC chains for focal parameters in the three primary models. For each model, traces are shown for the parameter most directly tied to the main biological inference and for the phylogenetic random-effect variance from one representative tree-level fit. Distinct colours denote the four independent chains. Well-behaved traces should overlap broadly among chains, fluctuate around a stable mean, and show no persistent trends, abrupt shifts, or long-term separation, all of which would suggest poor mixing or incomplete convergence.

Table S7. Gelman-Rubin convergence diagnostics (PSRF) by model across tree-level fits. Reported values include the median and maximum multivariate PSRF (mPSRF), together with the median and worst per-parameter PSRF among monitored parameters. Values close to 1 indicate good agreement among chains, whereas persistent values above approximately 1.1 suggest that at least some parameters may not have mixed fully.

| Model | Tree-level fits | Median mPSRF | Maximum mPSRF | Median worst PSRF | Worst PSRF observed | Parameters with PSRF > 1.1 |
| --- | --- | --- | --- | --- | --- | --- |
| 001 | 50 | 1.010 | 1.087 | 1.011 | 1.077 | 0 |
| 002 | 50 | 1.008 | 1.064 | 1.007 | 1.062 | 0 |
| 003 | 25 | 1.024 | 1.072 | 1.024 | 1.062 | 0 |
| 004 | 25 | 1.034 | 1.143 | 1.036 | 1.136 | 1 |
| 005 | 50 | 1.016 | 1.100 | 1.017 | 1.104 | 1 |
| 006 | 25 | 1.033 | 1.101 | 1.036 | 1.096 | 0 |
| 007 | 25 | 1.031 | 1.109 | 1.029 | 1.100 | 1 |
| 008 | 25 | 1.029 | 1.086 | 1.027 | 1.091 | 0 |
| 009 | 25 | 1.013 | 1.088 | 1.012 | 1.081 | 0 |
| 010 | 25 | 1.020 | 1.065 | 1.019 | 1.058 | 0 |
| 011 | 25 | 1.034 | 1.124 | 1.034 | 1.122 | 1 |
| 012 | 25 | 1.026 | 1.125 | 1.025 | 1.121 | 1 |
| 013 | 25 | 1.031 | 1.110 | 1.036 | 1.112 | 2 |
| 014 | 25 | 1.034 | 1.166 | 1.029 | 1.154 | 2 |
| 015 | 25 | 1.023 | 1.092 | 1.025 | 1.085 | 0 |
| 016 | 25 | 1.038 | 1.129 | 1.037 | 1.122 | 3 |
| 0NA | 756 | 1.028 | 1.469 | 1.028 | 1.744 | 40 |

Table S8. Effective sample size (ESS) diagnostics by model across all archived chain files. For each chain, ESS was calculated for all fixed and variance parameters, and the summary is based on the minimum ESS within that chain (that is, the worst-sampled parameter). Higher ESS indicates greater Monte Carlo precision, whereas counts below 200 and 500 provide conservative warning thresholds for potentially under-sampled chains.

| Model | Archived chains | Minimum chain ESS | Median chain ESS | 10th percentile ESS | Chains with ESS < 200 | Chains with ESS < 500 |
| --- | --- | --- | --- | --- | --- | --- |
| 001 | 200 | 38.5 | 135.2 | 62.7 | 181 | 200 |
| 002 | 200 | 41.9 | 130.4 | 58.1 | 185 | 200 |
| 003 | 100 | 25.8 | 45.5 | 35.9 | 100 | 100 |
| 004 | 100 | 26.0 | 48.2 | 36.4 | 100 | 100 |
| 005 | 200 | 21.7 | 71.8 | 33.3 | 200 | 200 |
| 006 | 100 | 17.8 | 40.0 | 31.5 | 100 | 100 |
| 007 | 100 | 29.8 | 43.5 | 34.9 | 100 | 100 |
| 008 | 100 | 22.6 | 41.4 | 31.8 | 100 | 100 |
| 009 | 100 | 45.5 | 76.5 | 60.1 | 100 | 100 |
| 010 | 100 | 44.0 | 75.8 | 59.5 | 100 | 100 |
| 011 | 100 | 22.9 | 46.4 | 35.2 | 100 | 100 |
| 012 | 100 | 21.2 | 45.8 | 33.9 | 100 | 100 |
| 013 | 100 | 20.2 | 38.2 | 29.8 | 100 | 100 |
| 014 | 100 | 24.6 | 41.7 | 32.1 | 100 | 100 |
| 015 | 100 | 24.8 | 42.0 | 31.4 | 100 | 100 |
| 016 | 100 | 24.2 | 42.9 | 33.8 | 100 | 100 |
| 0NA | 3,012 | 7.4 | 45.6 | 32.2 | 3,012 | 3,012 |


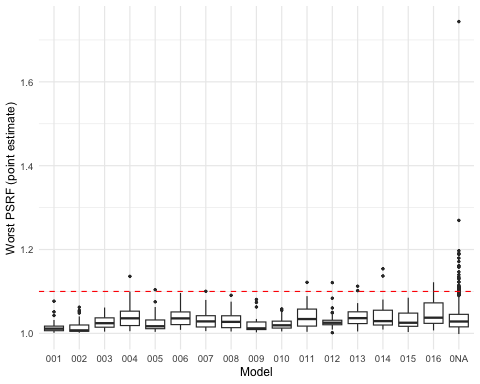


Figure S2. Distribution of worst per-parameter Gelman-Rubin potential scale reduction factors (PSRF) across phylogenetic trees for each model. For each tree-level fit, the plotted value is the largest PSRF among monitored parameters, so each point in the distribution reflects the least well-mixed parameter for that fit. Values close to 1 indicate good convergence among chains, whereas values approaching or exceeding the dashed 1.1 threshold indicate that at least one parameter may require more sampling.
